# Assessment interocular symmetry of peripapillary vessel density in young myopes with optical coherence tomographic angiography

**DOI:** 10.1007/s10792-023-02737-8

**Published:** 2023-07-29

**Authors:** Lin Liu, Man-li Deng, Min Li, Ding Xu, Le Feng, Jun Zou, Fang Wang

**Affiliations:** 1 Department of Ophthalmology, Clinical Medical College of Shanghai Tenth People’s Hospital of Nanjing Medical University, Shanghai, 200072 China; 2 Department of Ophthalmology, Shanghai Tenth People’s Hospital, Tongji University School of Medicine, 301# Yan Chang Middle Road, Shanghai, 200072 China

**Keywords:** Interocular symmetry, Vessel density, Myopia

## Abstract

**Purpose:**

The purpose of our study was to evaluate the interocular symmetry and distribution of peripapillary vessel density in young myopic eyes.

**Methods:**

A cross-sectional observational study was designed. A total of 174 eyes of 87 young myopic patients were recruited in this study. According to spherical equivalent (SE), 48 eyes were classified as mild myopia with a mean SE of − 2.12D (SD 0.66D), 66 as moderate myopia with a mean SE of − 4.50D (SD 0.87D), and 60 as high myopia with a mean SE of − 7.39D (SD 1.30D). Optical coherence tomography angiography (OCTA) was used to measure the vessel density. The distribution and interocular symmetry of peripapillary vessel densities were analyzed.

**Results:**

The vessel densities in the whole image, peripapillary, superior and inferior sectors were significantly lower in the high myopia group than in the mild or moderate myopia group (All *P* < 0.001), and the density in the nasal sector was significantly lower in the high myopia group than in the mild group. And most interesting, the vessel densities in the inside disc and temporal sector showed no difference among the three myopic groups (All *P* > 0.05). By Pearson correlation analysis, the vessel densities in the whole image, peripapillary, superior, inferior and nasal sectors were negatively correlated with axial length (AL) and SE (All *P* < 0.001), but vessel densities in the inside disc and temporal sector did not show this correlation (All *P* > 0.05). Interocular symmetry was observed in all the vascular parameters through paired-samples t-tests (All* P* > 0.05), intraclass correlation coefficient (ICC) and Pearson correlation analysis (All *P* < 0.001).

**Conclusion:**

The density of radial peripapillary capillaries decreased in the myopic eye with axial elongation, and optical vascular parameters showed significant interocular symmetry among young myopic eyes.

## Introduction

Myopia has become a major health problem around the world, particularly in East Asia, due to its complications that may cause blindness [1]. In myopic eyes, with the changes in the optic disc, parapapillary zone and ocular elongation axis, the retina may thin and less oxygen, leading to decreased blood circulation and retinal microvascular attenuation [2, 3]. So, retinal vascular morphology should be timely evaluated in myopic patients.

Interocular symmetry is an indicator to judge the pathological changes in eyes [4]. Asymmetry may occur between organ pairs during the progression of diseases [5]. Indeed, asymmetry between eyes is valuable for identifying pathological conditions, such as early glaucomatous damage [6]. Interocular asymmetry between retinal biometric parameters may be indicative of disease [7, 8]. However, the interocular symmetry of vessel density (VD) in myopia is not well characterized. Shahlaee et al. [9] found no significant differences in foveal avascular zone area (FAZA) between myopic patients and healthy controls. Hou et al. [6] reported that interocular asymmetry of vessel density can be quantified by OCT-A measurement, and glaucoma patients demonstrated significant vessel density asymmetry compared with healthy eyes. Therefore, interocular vessel density asymmetry can help identify pathological conditions.

Radial peripapillary capillaries (RPCs), comprising a unique network of capillary beds within a retinal nerve fiber layer (RNFL), provide nutrition for retinal ganglion cell axons. However, information is limited regarding interocular symmetry of peripapillary vessel density and distribution in myopia. Therefore, the current study was performed to evaluate the interocular symmetry and distribution of peripapillary vessel density in young myopic eyes.

## Materials and methods

### Subjects

We performed a cross-sectional observational study on myopic eyes of young Chinese. The study was approved by the Ethics Committee of Shanghai Tenth People’s Hospital (No: 20KT168) and was registered at Chinese Clinical Trial Registry (no: ChiCTR2000035768). A total of 174 eyes of 87 consecutive myopic candidates examined at Shanghai Tenth People’s Hospital were enrolled from September 2020 to August 2021. The median age was 25 years (range, 18–35 years). The average spherical equivalent (SE) was − 4.84 D (SD 2.31D, range − 0.5D to − 10.625D), and the average axial length (AL) was 25.56 ± 1.21 mm (range, 22.86 to 28.49 mm). According to SE, the subjects were divided into three groups: mild myopia group (− 0.5 D ≤ SE < − 3.0 D), moderate myopia group (− 3 D ≤ SE < -6.0 D and high myopia group (SE ≥ − 6.0 D). Myopia was mild in 48 eyes with a mean SE of − 2.12D (SD 0.66D), moderate in 66 eyes with a mean SE of − 4.50D (SD 0.87D) and high in 60 eyes with a mean SE of − 7.39D (SD 1.30D). None had a history of ocular surgery, ocular trauma, or systemic or ocular health abnormality. The demographic and ophthalmic characteristics of the three groups are summarized in Table 1. The study adhered to the tenets of the Declaration of Helsinki, and written informed consent was obtained by all subjects.

**Table 1:** Basic demographic and ophthalmic characteristics of three group (n = 174 eyes)

	Sex (male/female)	Median age (range, year)	SE (D)	Axial length (mm)	Intraocular pressure (mmHg)
Mild group	11/13	26 (18–35)	− 2.12 (0.66)	24.44 (0.69)	15.77 (2.62)
Moderate group	15/18	24 (18–34)	− 4.50 (0.87)	25.48 (0.77)	15.69 (1.67)
High group	14/16	25 (18–35)	− 7.39 (1.30)	26.56 (1.11)	16.13 (2.26)
χ2/F value	0.019	1.908	294.74	41.56	0.634
*P* value	0.990^*△*^	0.151^*^	0.000^*^	0.000^*^	0.227^*^

SE: spherical equivalent, *one-way ANOVA, *△ χ2* test

All eligible subjects were invited to undergo a comprehensive eye examination. Visual acuity, refraction and intraocular pressure (IOP) were examined by a noncontact tonometer; **AL** in each eye by the IOL Master 700® (Carl Zeiss Meditec AG, Jena, Germany); anterior segment by a slit lamp; fundus evaluated by slit-lamp biomicroscopy using Goldmann three-mirror contact lens and wide-field laser ophthalmoscopy (Optos, Marlborough, MA, UK). Exclusion criteria included a history of prior vitreous or retinal surgery, anisometropic eye, pathologic myopia, the glaucoma subject and glaucoma suspect, evidence of retinal disease affecting the retina or optic nerves by examination and systemic diseases potentially affecting the eyes. Measurements were performed from 8:00 to 11:30 to minimize the potential impact of diurnal variation.

### Optical coherence tomography angiography

The Optovue Angiovue software (Optovue, Inc. version 2015.1.1.98) was used for characterizing the vascular structures of the retina at the capillary level. The optic disc OCTA scan was performed as volumetric scans generally covering an area of 4.5 × 4.5 mm^2^ centered around the optic disc. Whole enface image vessel density in optic nerve head (ONH) was measured in the entire 4.5 × 4.5 mm^2^ image, and circumpapillary vessel density was calculated in a 750-µm-wide elliptical annulus extending from the optic disc boundary. All the measurements were taken by one well-trained investigator (author LL). Studies have reported that vessel density was lower on OCTA images with weaker signals [10–12], so only images with scan quality of 6 or greater and without significant motion artifacts were included in the study. The measurements were made for each patient in the OCTA according to the mean of values acquired for two times.

### Statistical analysis

Statistical analyses were performed using SPSS V.17.0 software (SPSS Inc, Chicago, IL, USA).

The non-normally distributed data were analyzed by one-sample K–S test. The continuous variables (SE, AL and **IOP**) were compared among three groups with one-way analysis of variance (except gender compared by Chi-square tests). One-way analysis of variance with the post hoc Bonferroni correction was performed to compare vessel density parameters among three groups and differences between each pair of groups. The correlation between vessel density and AL/SE was measured by the Pearson correlation. Interocular symmetry was evaluated using paired-samples t-test, Pearson’s correlation coefficient and ICC values. P value of less than 0.05 was considered statistically significant.

## Results

### Basic demographic and ophthalmic characteristics

A total of 184 eyes of 92 subjects were initially enrolled. Five subjects were excluded due to poor-quality OCTA images. The remaining 174 eyes of 87 subjects were included, consisting of 48 eyes from 24 subjects in the mild myopia group (− 0.5 ≤ SE < -3.0 D; mean − 2.12 ± 0.66D), 66 eyes from 33 subjects in the moderate myopia group (− 3.0 ≤ SE < -6.0 D; mean − 4.50 ± 0.87 D) and 60 eyes from 30 subjects in the high myopia group (SE ≥ -6.0 D; mean − 7.39 ± 1.30 D). The basic demographics and ocular characteristics are shown in Table 1. There were significant differences among three groups in **AL** and **SE** (all *P* < 0.01), but not in age, gender and **IOP** (all *P* value > 0.05). The high myopia group had a longer AL (24.44 ± 0.69 mm, 25.48 ± 0.77 mm and 26.56 ± 0.11 mm in the mild myopia, moderate myopia and high myopia groups, respectively, *P* < 0.01).

### Vessel density and distribution in three groups

The vessel densities in optic nerve heads are presented in Table 2. The whole-image RPC density in the high myopia group was 47.88%, significantly lower than those in mild myopia group (50.14%) and moderate myopia group (49.26%). The whole-image RPC density tended to decrease with the aggravation of myopia.

**Table 2:** Comparison of vessel density of optic nerve head among three groups (%)

	Mild group (*n* = 48)	Moderate group (*n* = 66)	High group (*n* = 60)	Average	P	Multiple comparisons
Whole image	50.14 (1.52)	49.26 (2.26)	47.88 (2.67)	49.02 (2.42)	0.000	Mild/Moderate > High
Inside disc	55.27 (4.03)	55.29 (4.30)	54.26 (4.34)	51.04 (3.02)	0.999	
Peripapillary	52.17 (1.74)	51.56 (3.00)	49.58 (3.30)	51.04 (3.02)	0.000	Mild/Moderate > High
Superior	54.02 (2.96)	53.06 (4.14)	50.45 (4.07)	52.43 (4.08)	0.000	Mild/Moderate > High
Temporal	55.42 (2.89)	55.76 (2.77)	54.72 (3.56)	55.30 (3.11)	0.197	
Inferior	53.98 (2.22)	53.47 (3.78)	51.00 (3.50)	52.76 (3.54)	0.000	Mild/Moderate > High
Nasal	47.73 (1.83)	5 46.08 (4.13)	444.70 (4.62)	5 46.06 (4.00)	0.000	Mild > High

**P**: one-way ANOVA, multiple comparisons: Bonferroni correction

**Fig. 1:**
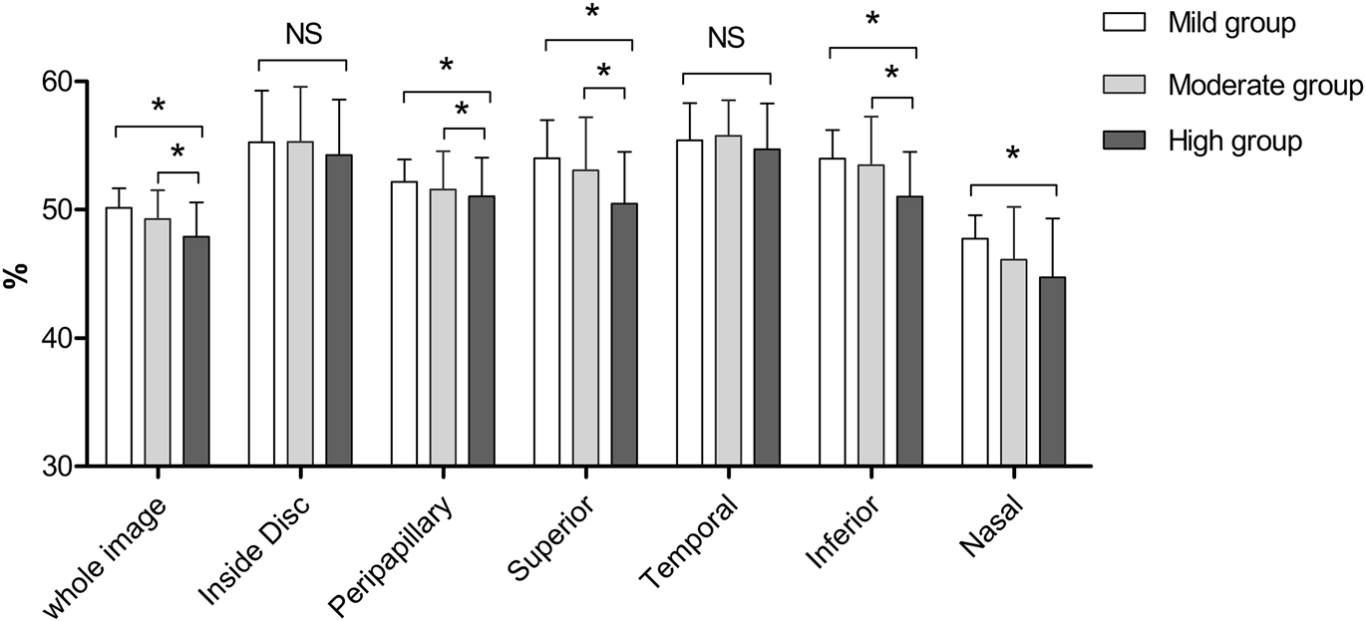
Comparison of vessel density of optic nerve head among three groups. *p < 0.001 NS: no significance

We further clarified the distribution of vessel density around the optic disc. The vessel densities in the peripapillary, superior and inferior sectors were significantly lower in the high myopia group compared with those in the mild or moderate myopia group, and the vessel density in the nasal sector was significantly lower in the high myopia group than in the mild group (All *P* value < 0.001). Surprisingly, the vessel density in the inside disc and temporal sector showed no difference among the three groups (All *P* value > 0.05) (Table 2, Fig. 1).

### Correlations of vascular parameters with AL and SE

Pearson’s correlation analyses were conducted to investigate the associations between vascular parameters and ocular variables in all study eyes. By analysis, the whole and peripapillary vessel densities were negatively correlated with AL (r = − 0.506, *P* < 0.001, r = − 0.423, *P* < 0.001; respectively), while the vessel density in the inside disc did not show this correlation (r = − 0.068, *P* = 0.374). Among different sectors, a significantly negative correlation was observed between AL and peripapillary vessel density in the superior sector (r = − 0.392,* P* < 0.001), inferior sector (r = − 0.447, *P* < 0.001) and nasal sector (r = − 0.334,* P* < 0.001). In contrast, AL showed no association with the vessel density in the temporal sector (r = 0.110, *P* = 0.149) (Table 3).

**Table 3:** Correlation analyses between axial length/SE and vessel density (Pearson analysis. *n* = 174 eyes)

	Axial length		SE	
	r_1_	*P*_*1*_	r_2_	*P*_*2*_
Whole image	− 0.506	0.000	− 0.453	0.000
Inside disc	− **0.068**	**0.374**	− **0.029**	**0.706**
Peripapillary	− 0.423	0.000	− 0.411	0.000
Superior	− 0.392	0.000	− 0.410	0.000
Temporal	− **0.110**	**0.149**	− **0.062**	**0.415**
Inferior	− 0.447	0.000	− 0.393	0.000
Nasal	− 0.334	0.000	− 0.316	0.000

Similar results were found between SE and vessel density. The whole and peripapillary vessel densities were negatively correlated with SE (r = − 0.453, *P* < 0.001, r = − 0.411, *P* < 0.001; respectively). There was no correlation between the vessel density in the inside disc and SE (r = − 0.029, *P* = 0.706). As expected, the SE was strongly and inversely associated with the peripapillary vessel density in the superior sector (r = − 0.410, *P* < 0.001), inferior sector (r = − 0.393, *P* < 0.001) and nasal sector (r = − 0.316, *P* < 0.001). However, we did not observe any correlation between the vessel density in the temporal sector and SE (r = 0.062, *P* = 0.415) (Table 3).

### Interocular symmetry of vessel density parameters in optic nerve heads

Table 4 summarizes the interocular symmetry of SE, AL and VD. There was no significant interocular difference in all the three parameters among three groups through paired-samples t-tests (All *P* value > 0.05). Pearson’s correlation analysis showed high interocular symmetry of VD in three groups (All *P* value < 0.01). In addition, the interocular correlation coefficients (ICCs) verified the significant symmetry of the vessel density in the whole image, inside disc, peripapillary and four sectors (*P* < 0.001 in all ICC values). All the evidence suggests that vessel density parameters of optic nerve heads show interocular symmetric features.

**Table 4:** Interocular symmetry of vessel density in optic nerve head (n = 87 subjects)

	Vessel density(%)			Measure of symmetry			
	Right eye	Left eye	*P1*	Interocular correlation	ICC	*P2*	*P3*
*Mild group (n* = *24)*							
SE	− 2.11 (0.71)	− 2.13 (0.61)	0.783	0.812	0.802	< 0.001	< 0.001
AL	24.44 (0.72)	24.45 (0.68)	0.703	0.963	0.961	< 0.001	< 0.001
Whole image	50.17 (1.62)	50.11 (1.45)	0.771	0.805	0.800	< 0.001	< 0.001
Inside disc	55.33 (3.92)	55.20 (4.22)	0.783	0.836	0.834	< 0.001	< 0.001
Peripapillary	52.20 (1.83)	52.14 (1.69)	0.716	0.906	0.903	< 0.001	< 0.001
Superior	53.85 (2.73)	54.19 (3.21)	0.417	0.791	0.780	< 0.001	< 0.001
Temporal	55.67 (2.99)	55.17 (2.84)	0.223	0.776	0.775	< 0.001	< 0.001
Inferior	53.79 (2.04)	54.17 (2.40)	0.185	0.830	0.818	< 0.001	< 0.001
Nasal	47.92 (1.69)	47.54 (1.98)	0.195	0.729	0.720	0.006	< 0.001
*Middle group (n* = *33)*							
SE	− 4.56 (0.88)	− 4.44 (0.88)	0.122	0.882	0.882	< 0.001	< 0.001
AL	25.50 (0.76)	25.45 (0.79)	0.291	0.938	0.937	< 0.001	< 0.001
Whole image	49.17 (2.23)	49.35 (2.33)	0.358	0.880	0.879	< 0.001	< 0.001
Inside disc	55.12 (4.12)	55.47 (4.53)	0.475	0.799	0.796	< 0.001	< 0.001
Peripapillary	51.45 (3.20)	51.66 (2.83)	0.500	0.847	0.840	< 0.001	< 0.001
Superior	52.82 (4.56)	53.30 (3.72)	0.397	0.711	0.696	< 0.001	< 0.001
Temporal	55.73 (2.54)	55.79 (3.02)	0.868	0.734	0.723	< 0.001	< 0.001
Inferior	52.12 (4.12)	53.82 (3.42)	0.127	0.786	0.772	< 0.001	< 0.001
Nasal	45.97 (4.17)	46.18 (4.16)	0.686	0.743	0.743	< 0.001	< 0.001
*High group (n* = *30)*							
SE	− 7.43 (1.26)	− 7.34 (1.36)	0.519	0.857	0.854	< 0.001	< 0.001
AL	26.58 (1.14)	26.53 (1.10)	0.354	0.968	0.967	< 0.001	< 0.001
Whole image	47.71 (2.51)	48.04 (2.92)	0.370	0.754	0.745	< 0.001	< 0.001
Inside disc	55.44 (4.79)	55.11 (5.15)	0.637	0.712	0.710	< 0.001	< 0.001
Peripapillary	49.66 (3.18)	49.50 (3.48)	0.767	0.614	0.612	< 0.001	< 0.001
Superior	50.33 (4.37)	50.57 (3.83)	0.676	0.735	0.729	< 0.001	< 0.001
Temporal	54.77 (3.66)	54.32 (3.64)	0.604	0.670	0.665	< 0.001	< 0.001
Inferior	51.09 (4.01)	50.90 (3.60)	0.882	0.611	0.606	< 0.001	< 0.001
Nasal	44.63 (4.60)	45.03 (5.01)	0.618	0.639	0.639	< 0.001	< 0.001

P1: the paired t-test; P2: Pearson correlation analysis; P3: intraclass correlation coefficient

ICC, interclass correlation coefficient. AL: axial length, SE: spherical equivalent

## Discussion

In our study, we evaluated the **RPC** density that directly supplies the retinal ganglion cells in young myopic individuals. To reduce the effect of **AL** and SE, anisometropic eyes were excluded. Vessel density displayed evident interocular symmetry in three myopic groups. Interocular vessel density asymmetry may serve as a complementary parameter to assess retinal disorders. OCTA allows noninvasive visualization and quantification of the retinal vasculature without the use of exogenous intravenous dye injection [13]. Reproducible quantitative tools have emerged to assess the vessel density around the optic disc, including in the optic nerve head and **RPC** layer [10, 14]. This quantification can provide detailed information about retinal vascular circulation in the treatment of subclinical retinal and optic diseases [15].

Previous studies using OCTA have revealed vascular changes in myopia may be related to axial elongation. Using OCTA, Wang et al*.* found a significant reduction in RPC flow density in severe myopic eyes, compared with that in emmetropic eyes [16]. Further, the peripapillary vessel density drops in myopia patients, and much in those with myopic glaucoma [2]. Yaprak AC et al*.* have reported that the whole and peripapillary vessel densities were significantly decreased in the high myopia group but did not observe any correlation between AL and inside-disc vessel density [17]. Consistently to previous reports, we found an obvious decrease in vessel density in all regions of the RPC area in high myopia group eyes than in the mild myopia group, except that in the inside disc and temporal sector. OCT angiography has shown that high myopia can decrease retinal perfusion in the peripapillary region, compared with the mild myopia.

In addition, we found that RPC vessel density was negatively related to the AL and myopia diopter, but in the inside disc and temporal sector, RPC vessel density was not markedly associated with refractive error and axial length. In other words, with the increase of AL or myopia diopter, RPC vessel density decreased in most sectors of the peripapillary region, except for the inside disc and temporal sector. These findings indicate that retinal vascular dropout in the myopic process may be related to the mechanical axial elongation.

Since the eyeball in a myope is prolate and may further stretch and thin the retina and shift the optic nerve. The optic nerve head deformation may lead to retinal structural alterations in myopic eyes [18]. Myopic eyeball elongation decreases retinal function and oxygen consumption, as shown by the low blood circulation and retinal capillary loss in high myopia [3, 19]. A decrease in the vessel density may lead to hypoxia and nutritional deficiency in the retina and optic nerves, which may help to explain the relationship between longer AL and various pathologic changes in high myopia.

Interestingly, in our study, the vessel densities in all regions decreased with AL and SE, except those in the inside optic disc and temporal sector. So, we consider that among all the vascular parameters, the flow in the inside optic disc and temporal sector flow is not associated with retinal capillary loss related to myopia. Therefore, myopia can lead to the density of retinal capillary decrease in these peripapillary parts (such as superior, inferior and nasal sectors). Any abnormal vessel density in the inside optic disc and temporal sector can indicate the presence of pathologic disorders. The reduced vessel density could be related to the reduced blood supply around the optic disc, primarily due to mechanical stretching of the corresponding regions. Various studies have investigated the impact of myopic tilted disc on structural change of optic nerve. Fan et al. found that eyes with torted disc exhibited thicker temporal retinal and RNFL and more temporally positioned superior peak of RNFL [20], and during the myopic shift, the tilting and rotation of optic disc may be accompanied by nasal bulging and kinking of retinal nerve fibers [21]. Therefore, it could result in vessel density of the inside optic disc and temporal quadrant does not descend.

Healthy organ pairs mostly show symmetric anatomic features. Interocular asymmetry is observed in the condition of clinical diseases [4]. For instance, glaucoma often shows asymmetric features, particularly in the early stage [4, 8]. Several researchers have reported the symmetry of the retinal structure measured by OCT. By swept-source optical coherence tomography (SS-OCT), Lee SY et al. have found significant asymmetry of macular inner retinal layers between the glaucoma and normal groups [4]. Kim et al*.* have detected the symmetrical choroidal thickness of both eyes [22]. Using spectral-domain optical coherence tomography (SD-OCT), Field MG et al*.* have found that interocular retinal and RNFL thickness asymmetry are efficient to predict early glaucomatous damage [23]. Using OCTA, Shahlaee et al. have found no interocular asymmetry of FAZA among healthy subjects [9]. Glaucoma can significantly increase interocular asymmetry of vessel density in optic nerve head [6]. Our data prove that there were no significant differences between right eyes and left eyes among three myopic group, and vessel density display a high degree of interocular symmetry in three myopic groups even among patients with high myopia. The founding has not been reported before. Hereby by that means, in the case of similar diopters, the peripapillary vessel density is abnormally decreased in one eye, pathological disease should be considered. However, if the changes occurred in both eye, other factors can first be eliminated, such as refractive factors, which could influence the measurements by the OCTA, especially among high myopia patients, inter-eye vessel density asymmetry values may serve as a complementary parameter that can be assessed with other clinical parameters.

The major limitation of this study was that the change in optic nerve head parameters were parallel in this study, and longitudinal studies are needed to better characterize the relationship of vessel density with the development and progression of myopia. Additionally, we sought to eliminate the effect of age, a high proportion of young myopes were included, further studies with a larger age spectrum should be performed to evaluate the changes in the retinal capillaries.

In conclusion, myopic eyes display a high degree of interocular symmetry of optical vascular parameters. Asymmetry analysis can be a potential ancillary tool for diagnosing unilateral pathology that results in subtle asymmetrical capillary dropout in young myopic patients.

## Data Availability

All data generated during this study are included in this article. Further enquiries can be directed to the corresponding author.
